# A comparative study of evaluating missing value imputation methods in label-free proteomics

**DOI:** 10.1038/s41598-021-81279-4

**Published:** 2021-01-19

**Authors:** Liang Jin, Yingtao Bi, Chenqi Hu, Jun Qu, Shichen Shen, Xue Wang, Yu Tian

**Affiliations:** 1 Drug Metabolism and Pharmacokinetics, AbbVie Bioresearch Center, Worcester, MA 01605 USA; 2 Discovery and Exploratory Statistics, AbbVie Bioresearch Center, Worcester, MA 01605 USA; 3 Department of Pharmaceutical Science, SUNY at Buffalo, Buffalo, NY 14228 USA; 4 Center of Excellence in Bioinformatics & Life Science, Buffalo, NY 14203 USA

**Keywords:** Data processing, Proteomics, Proteome informatics

## Abstract

The presence of missing values (MVs) in label-free quantitative proteomics greatly reduces the completeness of data. Imputation has been widely utilized to handle MVs, and selection of the proper method is critical for the accuracy and reliability of imputation. Here we present a comparative study that evaluates the performance of seven popular imputation methods with a large-scale benchmark dataset and an immune cell dataset. Simulated MVs were incorporated into the complete part of each dataset with different combinations of MV rates and missing not at random (MNAR) rates. Normalized root mean square error (NRMSE) was applied to evaluate the accuracy of protein abundances and intergroup protein ratios after imputation. Detection of true positives (TPs) and false altered-protein discovery rate (FADR) between groups were also compared using the benchmark dataset. Furthermore, the accuracy of handling real MVs was assessed by comparing enriched pathways and signature genes of cell activation after imputing the immune cell dataset. We observed that the accuracy of imputation is primarily affected by the MNAR rate rather than the MV rate, and downstream analysis can be largely impacted by the selection of imputation methods. A random forest-based imputation method consistently outperformed other popular methods by achieving the lowest NRMSE, high amount of TPs with the average FADR < 5%, and the best detection of relevant pathways and signature genes, highlighting it as the most suitable method for label-free proteomics.

## Introduction

Large-scale protein quantitation of biospecimens is critical for biological, biomedical and pharmaceutical researches. Liquid chromatography-mass spectrometry (LC–MS) based proteomics technology is a popular approach in profiling global protein expressions. Thousands of proteins can be identified and quantified in a single MS injection. Two common quantification methods have been developed in bottom-up proteomics: label-based and label-free^1^. Label-free proteomics is more commonly employed in large-scale biological studies because of its cost-effective sample processing and capacity of quantifying a very large number of samples in one batch.

Key applications of label-free proteomics include the discovery of biomarkers and new drug targets, but a major issue is that the power of statistical inference and downstream functional analysis is greatly impacted by the presence of missing values (MVs) in the protein abundance data. Multiple factors contribute to MVs in LC–MS based proteomics, including biological factors, such as proteins do not exist and protein abundances are below the instrument detection limit, as well as analytical factors, such as sample loss in preparation, mis-cleavage of peptides during digestion, poor ionization efficiency and bad peptide-spectrum matches^2^. Missing values in proteomic data can be generally characterized into missing at random (MAR) and missing not at random (MNAR)^2,3^. MAR missing values mostly result from technical limitations and stochastic fluctuations in an abundance-independent manner, and oppositely, MNAR missing values are more abundance-dependent that can be explained by the measurability of the corresponding peptides. Missing values in proteomic data are a mixture of MAR and MNAR. Although the real proportion is difficult to determine, it is believed that MNAR plays a dominant role in producing missing values^4^.

To minimize the constraints of MVs, imputation methods are used to replace missing values with estimated values. Skewed distribution of protein abundances is often observed in proteomic data because of the existence of MNAR missing values^2^. Therefore, left-censored methods are applied as they replace MVs with small values closed to or smaller than the globally minimum quantitative value to mimic MNAR^3–6^. Imputation methods that are commonly utilized in other biological expression data (microarray, etc.)^7,8^, such as local similarity methods and global structure methods, have also been introduced to proteomics because they can handle mixed types of MVs^3,5^.

To date, what is the best method to handle MVs in proteomic data remains controversial. One big challenge is the lack of a high-quality, large-scale dataset to benchmark protein abundance that enables a more systematic and comprehensive evaluation. Proteomic datasets with spike-in controls were used in some evaluation studies but they either were relatively small-scale or contained only a few spike-in proteins^5,6^, which were not comparable to real-world proteomic data. Evaluation works using these datasets could be inaccurate and unreliable. Models were developed to generate artificial peptide and protein abundance for evaluating imputation methods^2,3^, but real proteomic data is still preferable because whether the simulated datasets bring systematic bias is not clear yet. Furthermore, the lack of a representative collection of imputation methods in one study also prevents the selection of the best method. For example, the top-ranked imputation methods from one study^5^ were not included in the other^3^, resulting in difficult cross comparisons. In addition, simulated missing values have been commonly used for evaluating the performance of imputation^3,9,10^, but testing with real missing values is still highly desirable. However, without knowing the real values, differences between imputed and real values cannot be calculated, thus a better strategy of comparison is needed.

A novel MS1-based label-free proteomics workflow, named IonStar, was developed recently to generate high-quality quantitative proteomic data for large-scale studies. It remarkably outperforms other popular proteomics software for its high quantitation accuracy and extremely low missing value rate^11^. In this study, we utilized a benchmark spike-in dataset generated by the IonStar workflow to explore the performance of imputation methods. Quantitative protein abundances were measured across 32 samples that were grouped with different portions of *E. coli* and yeast proteome spiked into the human proteome. Intragroup variations were added on purpose to mimic biological replicates of large-cohort studies. MAR and MNAR missing values were then incorporated with designated proportions to simulate different mechanisms of missingness. Protein ratios after imputation were compared with real spike-in ratios. Detection of differentially expressed proteins was also assessed in terms of true positives (TPs) and false altered-protein discovery rate (FADR). To allow an insightful yet not exhaustive comparison, we intentionally selected seven imputation methods: lowest of detection (LOD), random drawing from a left-censored normal distribution (ND), k-nearest neighbors (kNN), local least squares (LLS), random forest (RF), singular value decomposition (SVD) and Bayesian principal component analysis (BPCA). These methods cover left-censored, local similarity, and global-structure methods, and have demonstrated robust performance in previous studies^3,5,6,12,13^. Moreover, we utilized a label-free proteomic dataset of immune cells sorted by flow cytometry in this study. This dataset contains both steady and activated states of each cell type, and the alterations of signaling pathways regarding the activations have already been well understood^14^. Therefore, whether these pathways are detected after imputation can be used to evaluate the performance of imputing real missing values.

## Results

Two large-scale label-free proteomic datasets were selected to evaluate the performance of imputation methods. In the benchmark dataset, small and variable amounts of *E.coli* and yeast proteins were spiked into the human proteome with designated ratios. The proportion of human proteins in all samples remained constantly 70% as background. In group A to D, the proportion of *E.coli* proteins was 5%, 7.5%, 10%, and 12.5%, and the proportion of yeast proteins was 25%, 22.5%, 20%, and 17.5%, respectively. Therefore, between Group B-D and Group A, ratios of *E.coli* proteins were 1.5, 2, and 2.5, and ratios of yeast proteins were 0.9, 0.8, and 0.7 (Table S1). In addition, proportions of *E.coli* and yeast proteins were variable in eight individual samples of each group to mimic variations in biological replicates, while the mean values of each group were as designated and differences between Group B-D and Group A were statistically significant (p-value < 0.05) (Table S1). The benchmark dataset contains 1,184 *E.coli* proteins, 1,081 yeast proteins, and 4,424 human proteins without MVs. In another study, Rieckmann and colleagues profiled the proteomes of steady and activated states of 28 different types of blood cells sorted by flow cytometry from human donors^14^. The data of steady-state and activated B cells, T4 cells, T8 cells, and monocytes were selected for evaluating the performance of imputation methods. The immune cell dataset contains 10,081 proteins with 27% values are missing, within which 3,963 protein contain zero MVs.

**Figure 1:**
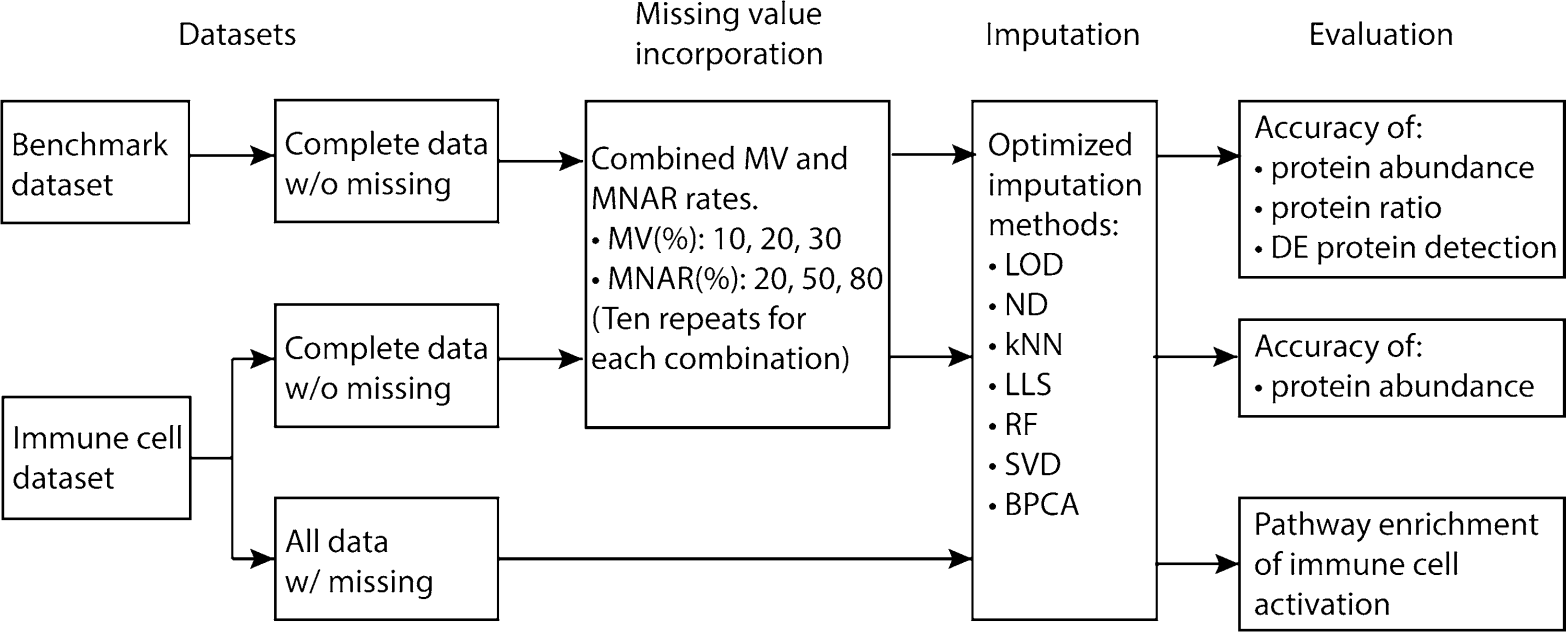
Analysis workflow. Missing values were incorporated into the benchmark dataset and complete part of the immune cell dataset in nine MV-MNAR combinations. Ten repeats were created for each MV-MNAR situation based on different random seeds. Datasets with missing values were imputed separately with seven methods: lowest of detection (LOD), random drawing from a left-censored normal distribution (ND), k-nearest neighbors (kNN), local least squares (LLS), random forest (RF), singular value decomposition (SVD) and Bayesian principal component analysis (BPCA). Accuracy of proteins abundances and intergroup protein ratios were compared based on normalized root mean square error (NRMSE) between real and after imputation. DE protein detection between Group B-D and Group A of the benchmark dataset was compared based on the amounts of true positives (TPs), false altered-protein discovery rate (FADR), and receiver operating characteristic (ROC) curves. The whole immune cell dataset with real missing values was imputed using seven methods, and enriched pathways of immune cell activation were compared.

To test the performance of imputation methods, simulated MVs were incorporated into the complete datasets at three different rates (10%, 20%, 30%). Three rates of MNAR (20%, 50%, 80%) were further combined with each MV rate to mimic low, middle, and high levels of MNAR. Ten dataset repeats were generated with the same missingness condition to test the reproducibility of imputation methods, resulting in ninety test datasets in total from each original dataset (Fig. 1). To ensure unbiased comparison, the key parameters of imputation methods were optimized. The parameters were tested with the benchmark dataset in three conditions (20%MV-20%MNAR, 20%MV-50%MNAR, 20%MV-80%MNAR). Normalized Root Mean Square Error (NRMSE) between imputed and real protein abundances was utilized to determine the accuracy of imputation. For the local similarity methods kNN and LLS, it is critical to select a k-value that determines the number of most similar proteins for estimation^15^. We chose k (number of nearest neighbors) = 6 for kNN and k (number of similar proteins used for regression) = 150 for LLS (Fig. S1A,B). The RF-based imputation method does not require tuning specific parameters^16^. The different number of trees to grow in each forest (ntree) did not show a significant change in imputation accuracy, thus default setting (ntree = 100) was applied (Fig. S1C). For global-structure methods, the accuracy of imputation largely depends on the number of principal components used for regression^17,18^. The number of principal components (nPCs) was set to 2 for both SVD and BPCA based on the overall best performance (Fig. S1D,E).

NRMSE has been widely used to evaluate the difference between imputed values and real values^9,10,18^. Therefore, we used NRMSE to compare the imputed and original protein abundances with both datasets (Fig. 1). Additional comparisons were performed with the benchmark dataset. First, the accuracy of the intergroup protein ratio was compared between the designated ratios and observed ratios after imputation by calculating NRMSE as well. Second, detection of DE proteins was compared by counting how many *E.coli* and yeast proteins (true positives, TPs) were significantly altered between Group B-D and Group A. Third, false altered-protein discovery rate (FADR), which was defined by the proportion of human proteins (false positives, FPs) in the total positives, was used to assess the false positive rate of DE protein detection after imputation^11^. The whole immune cell dataset consists of four cell types in steady and activated states with approximately 27% missing values. After imputation, Gene Ontology (GO) biological processes enrichment was analyzed with DE proteins from activation of each cell type to test if relevant proteins, activation processes and signaling pathways were detected (Fig. 1).

### Imputation accuracy of protein abundances and intergroup protein ratios with the benchmark datasets

**Figure 2:**
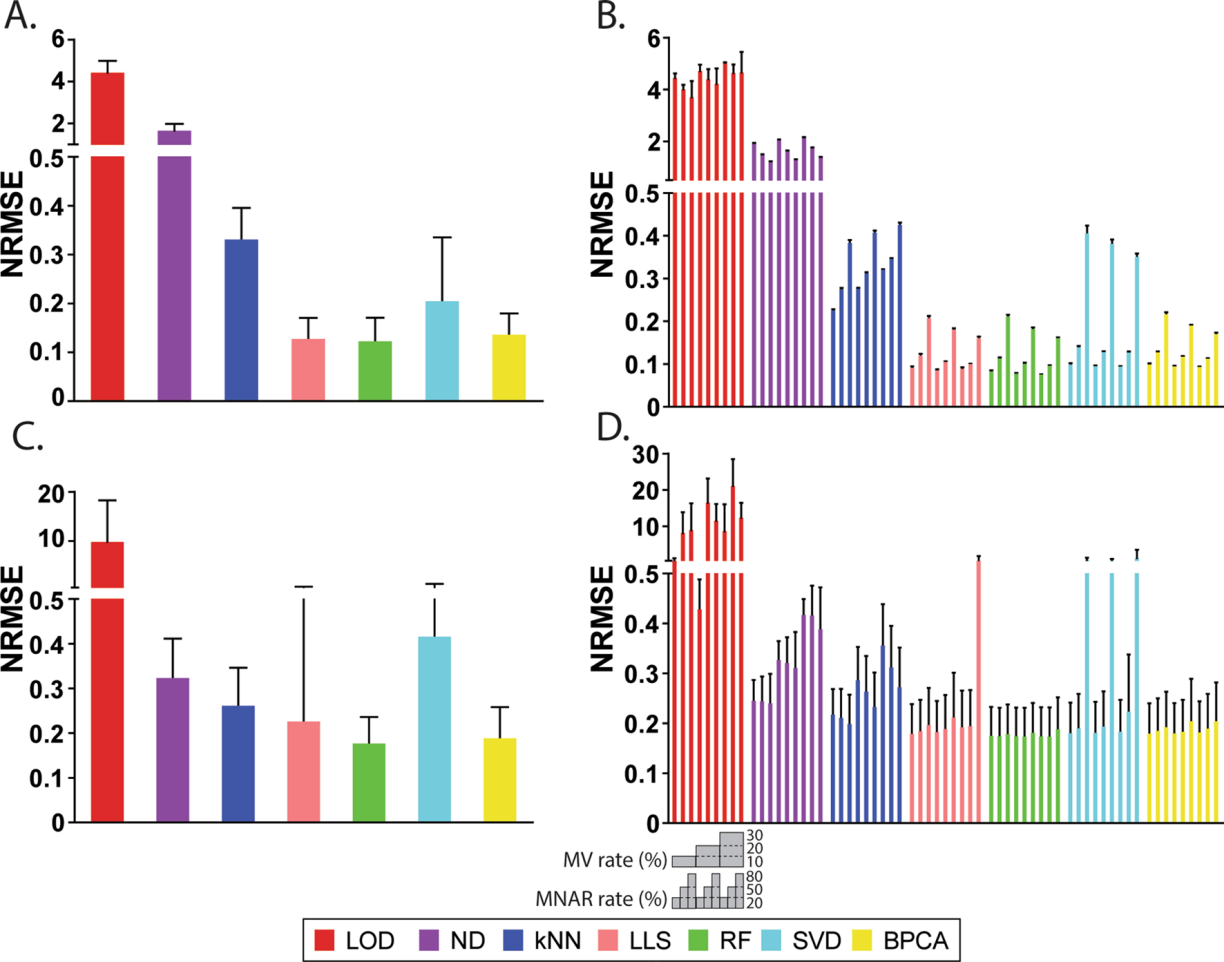
NRMSE of protein abundance and intergroup protein ratios with the benchmark dataset. (**A**) Overall NRMSE of protein abundances of seven imputation methods. (**B**) NRMSE of protein abundances in nine MV-MNAR conditions. (**C**) Overall NRMSE of intergroup protein ratios of seven imputation methods. (**D**) NRMSE of intergroup protein ratios in nine MV-MNAR conditions. In graph (**A**) and (**C**), bars represent average values of all ninety repeats, and error bars represent standard deviations. In graph (**B**) and (**D**), bars represent average values of ten repeats in each MV-MNAR condition, and error bars represent standard deviations. Nine MV-MNAR conditions are (from left to right): 10%MV-20%MNAR, 10%MV-50%MNAR, 10%MV-80%MNAR, 20%MV-20%MNAR, 20%MV-50%MNAR, 20%MV-80%MNAR, 30%MV-20%MNAR, 30%MV-50%MNAR, 30%MV-80%MNAR.

Accuracy of imputation was first evaluated by comparing NRMSE of imputed and original protein abundance values using the benchmark dataset. Overall, the left-censored methods, LOD and ND, had the highest average NRMSE of all test datasets, and RF and LLS estimated protein abundances with the highest accuracy as they displayed the lowest average NRMSE. Between the global-structure methods, BPCA performed better than SVD (Fig. 2A). Furthermore, NRMSE of protein abundances was compared in terms of different rates of MV and MNAR. When the MV rate increased, NRMSE of LOD, ND, and kNN increased as well. NRMSE of LLS, RF, SVD, and BPCA remained constant with low or middle level of MNAR regardless of the level of MV and slightly dropped when the MV rate increased with 80% MNAR in the datasets (Fig. 2B). Left-censored methods performed better with higher MNAR rates as expected. Interestingly, a remarkable boost of NRMSE was observed for non-left-censored methods when the MNAR rate increased from 50 to 80% (Fig. 2B). Particularly, NRMSE of SVD imputation displayed a larger increase between 50% MNAR and 80% MNAR than other methods, suggesting that SVD was the most sensitive method to high MNAR rate.

Besides of accuracy of protein abundance values, we also investigated whether relative protein ratios between groups could be accurately estimated after imputation. The protein ratios between Group B-D and Group A after imputation were compared with designated spike-in protein ratios and the differences were evaluated by NRMSE as well. RF and BPCA had the lowest average NRMSE of all test datasets and their NRMSE of protein ratios were consistent regardless of different rates of MV and MNAR (Fig. 2C,D), indicating that these two methods performed the best in estimating intragroup protein ratios. Of note, although LLS had similarly low level of NRMSE as RF in imputing protein abundances (Fig. 2A), it showed significantly higher NRMSE of protein ratios with extremely large variations among datasets (Fig. 2C), and the high NRMSE of LLS mostly resulted from 30%MV-80%MNAR datasets (Fig. 2D). Similar to LLS, SVD imputation performed better than ND and kNN in estimating protein abundances but had much higher NRMSE when estimating protein ratios (Fig. 2A,C) especially with datasets containing 80% MNAR missing values (Fig. 2D). Between the two left-censored methods, ND significantly outperformed LOD in estimating protein ratios with around 30-fold lower NRMSE. Together, these results suggest that RF imputation is the most accurate method in terms of protein abundance values and relative protein ratios, and the robustness of imputation is primarily affected by the rate of MNAR.

### Detection of differentially expressed proteins with the benchmark datasets

**Figure 3:**
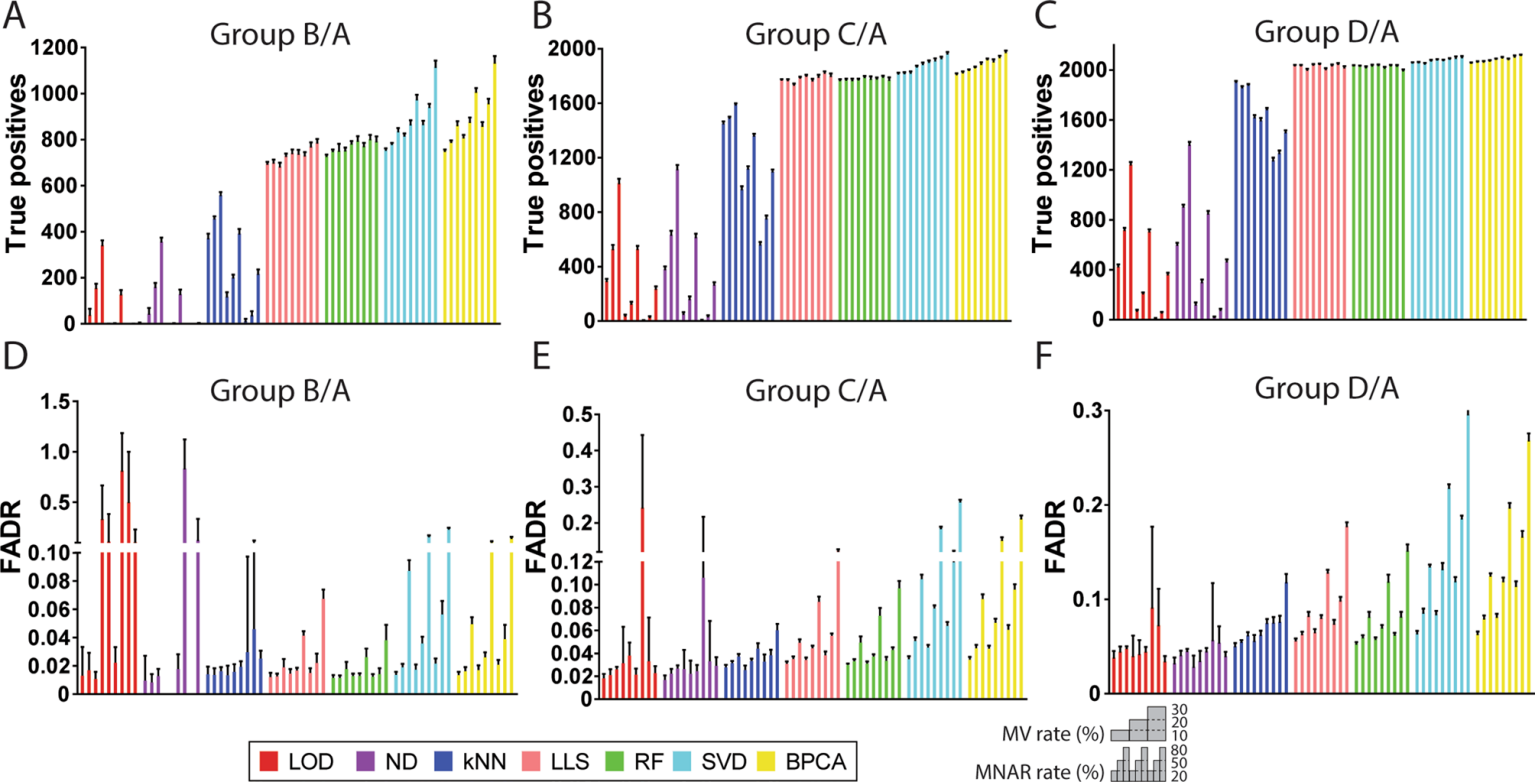
True positive detection and false altered-protein discovery rate with the benchmark dataset. Between Group B-D and Group A, true positives (TPs) are *E.coli* and yeast proteins with adjusted p-value < 0.05, and false positives (FPs) are human proteins with adjusted p-value < 0.05. False altered-protein discovery rate (FADR) is defined as the proportion of FPs in all positives. (**A**–**C**) Amounts of TPs detected in nine MV-MNAR conditions for each imputation method. (**D**–**F**) FADR in nine MV-MNAR conditions for each imputation method. In each graph, bars represent average values of ten repeats in each MV-MNAR condition, and error bars represent standard deviations. Nine MV-MNAR conditions are (from left to right): 10%MV-20%MNAR, 10%MV-50%MNAR, 10%MV-80%MNAR, 20%MV-20%MNAR, 20%MV-50%MNAR, 20%MV-80%MNAR, 30%MV-20%MNAR, 30%MV-50%MNAR, 30%MV-80%MNAR.

In the benchmark dataset, the spiked-in *E.coli* and yeast proteins were designed to be significantly different between Group B-D and Group A with intragroup variations. Therefore, the accuracy of DE protein detection can be assessed by the number of non-human proteins (TPs) that are significantly altered after imputation and the proportion of human proteins (FPs) in all significantly altered proteins (FADR). In general, imputation using LLS, RF, SVD, and BPCA detected more TPs than LOD, ND, and kNN (Fig. 3A–C). Between Group D and Group A, over 90% of non-human proteins displayed significant alteration after imputation by LLS, RF, SVD, and BPCA (Fig. 3C). Increasing of the MNAR rate or decreasing of the MV rate led to more TPs detected by LOD, ND, and kNN but less TPs detected by SVD and BPCA, while LLS and RF detected consistent amounts of TPs across different MV-MNAR conditions (Fig. 3A–C). Although SVD and BPCA appeared to detect more TPs than RF and LLS, especially in the comparison between Group B and Group A (Fig. 3A), it was notably that SVD and BPCA also introduced more FPs (Fig. 3D). The average FADR of RF and LLS (4.9% and 5.7%, respectively) were nearly half of those of SVD and BPCA (10.8% and 9.0%, respectively). Particularly for Group B/Group A comparison in datasets with 30%MV-80% MNAR, the average FADR of SVD was 23.8%, which was 6.3-fold higher than the average FADR of RF (3.8%) (Fig. 3D). Interestingly, although the amounts of TPs detected by LLS and RF imputation were somewhat constant with increasing MNAR, the FADR increased markedly. For example, in the situation of 20% MV in Group B/Group A comparison, RF and LLS detected 1% and 0% more TPs when the MNAR rate raised from 50 to 80%, respectively, but FADR of RF increased twofold and FADR of LLS increased 2.5-fold (Fig. 3A,D). The boost of FADR with a high MNAR rate was observed for most non-left-censored methods, including RF, LLS, SVD, and BPCA (Fig. 3D–F). These results suggest that the accuracy of DE protein detection of imputation methods is mainly determined by the rate of MNAR.

**Figure 4:**
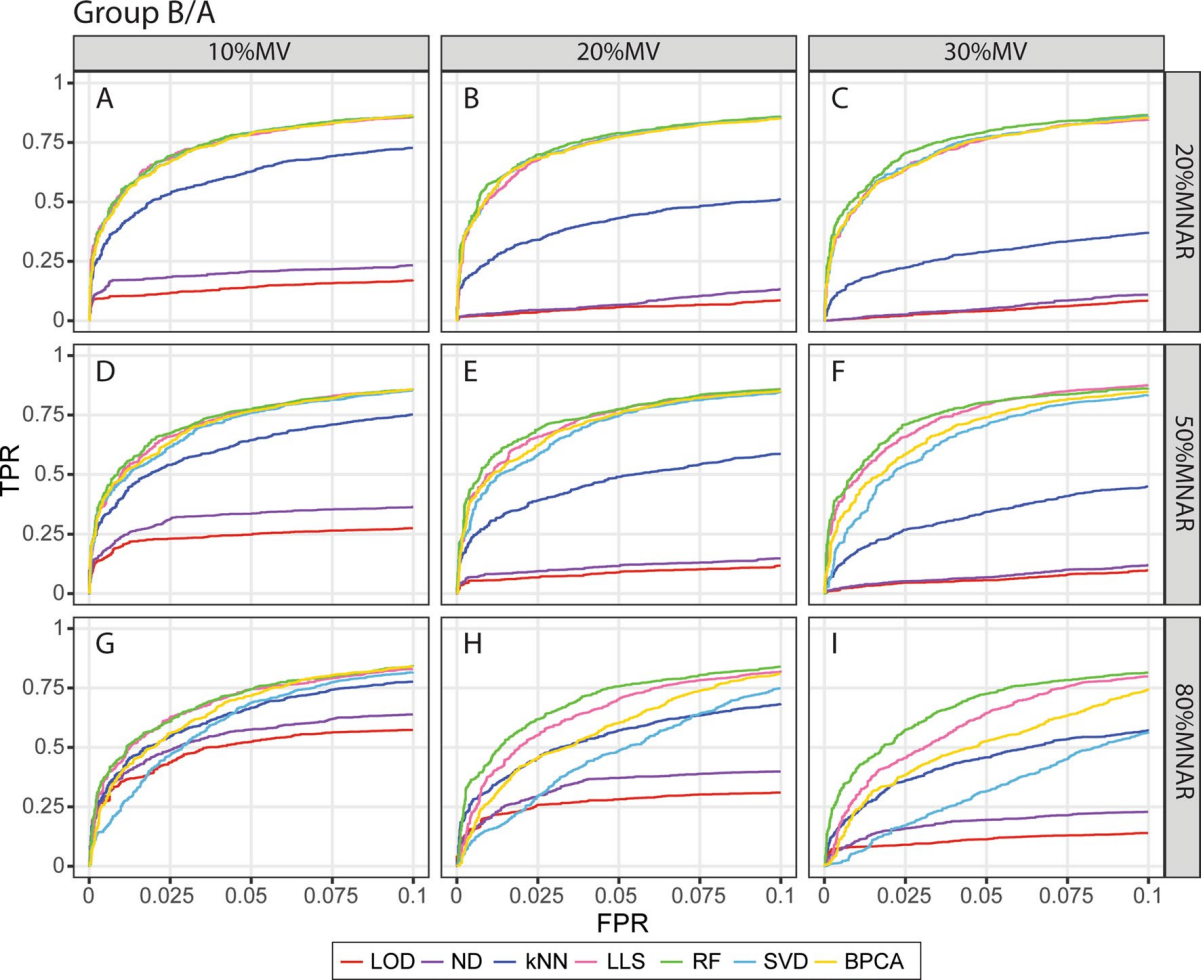
ROC curves of Group B/A with the benchmark dataset. ROC curves are plotted based on the average true positive rate (TPR) and false-positive rate (FPR) of ten repeats in each MV-MNAR condition. *E.coli* and yeast proteins are considered true positives and human proteins are considered false positives. (**A**–**I**): 10%MV-20%MNAR, 20%MV-20%MNAR, 30%MV-20%MNAR, 10%MV-50%MNAR, 20%MV-50%MNAR, 30%MV-50%MNAR, 10%MV-80%MNAR, 20%MV-80%MNAR, 30%MV-80%MNAR. This figure was generated with R 3.6.1 (https://www.r-project.org) package ggplot2 v 3.2.1 (https://ggplot2.tidyverse.org).

To further compare the accuracy of DE protein detection, we plotted the average true positive rate (TPR) and false-positive rate (FPR) on receiver operating characteristic (ROC) curves for different MV-MNAR conditions. For Group B/Group A comparison, RF, LLS, SVD, and BPCA explicitly demonstrated the best performance with 20% MNAR missing values (Fig. 4A–C). RF outperformed other methods by showing relatively higher TPR and lower FPR in most conditions with 50% and 80% MNAR (Fig. 4D–I). In the most extreme condition (30%MV-80%MNAR), RF achieved approximately 74% TPR with 5% FPR, which was roughly 10% higher than LLS and 20% higher than BPCA (Fig. 4I). Of note, the performance of SVD imputation was comparable with RF when the MNAR rate was low but dropped dramatically while the MNAR rate increased (Fig. 4), suggesting that the accuracy of SVD was largely dependent on the mechanism of missingness. Similar results were observed from the comparisons between Group C-D and Group A as well, that RF imputation performed equally well or better than other methods consistently (Fig. S2A,B). In summary, DE protein detection was the most accurate if RF imputation was employed.

### Evaluating imputation methods with the immune cell dataset

**Figure 5:**
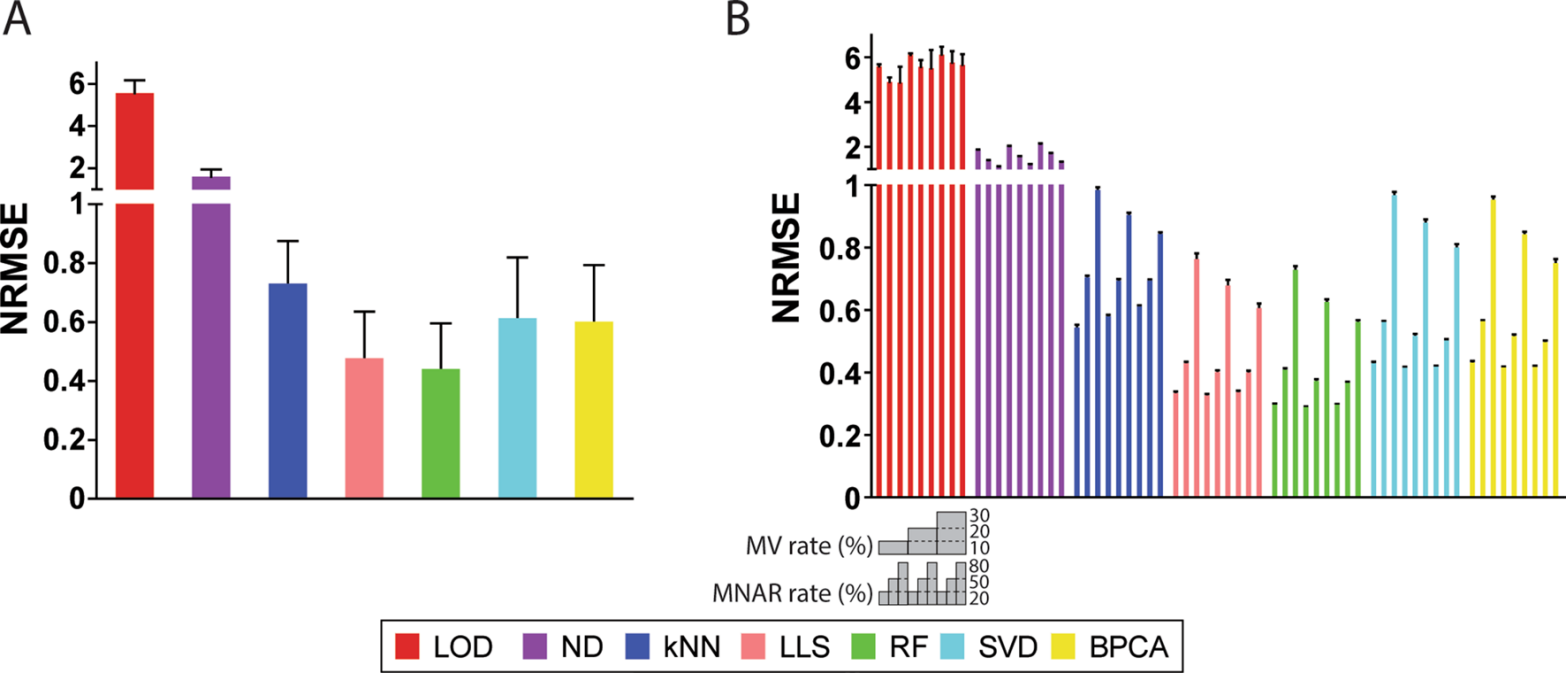
NRMSE of protein abundances with the immune cell dataset. (**A**) Overall NRMSE of protein abundances of seven imputation methods. Bars represent average values of all ninety repeats, and error bars represent standard deviations. (**B**) NRMSE of protein abundances in nine MV-MNAR conditions. Bars represent average values of ten repeats in each MV-MNAR condition, and error bars represent standard deviations. Nine MV-MNAR conditions are (from left to right): 10%MV-20%MNAR, 10%MV-50%MNAR, 10%MV-80%MNAR, 20%MV-20%MNAR, 20%MV-50%MNAR, 20%MV-80%MNAR, 30%MV-20%MNAR, 30%MV-50%MNAR, 30%MV-80%MNAR.

Accuracy of imputing protein abundance values was also evaluated using the complete part of the immune cell dataset, which contained 3,963 proteins. Missing values were incorporated with the same MV-MNAR conditions as described before for the benchmark dataset (Fig. 1). RF imputation again achieved the lowest average NRMSE of all test datasets while left-censored methods showed the highest average NRMSE (Fig. 5A). Unlike the benchmark dataset, with which SVD displayed much higher NRMSE than BPCA (Fig. 2A), SVD and BPCA had similar average NRMSE with the immune cell datasets (Fig. 5A). Furthermore, we compared the NRMSE across different MV-MNAR conditions. Accuracy of left-censored methods, LOD and ND, rose with the increase of MNAR rate and fell with the increase of MV rate. The non-left-censored methods shared similar patterns that increasing MV rate with 20% or 50% MNAR did not affect NRMSE much and increasing MV rate with 80% MNAR slightly reduced NRMSE (Fig. 5B). Additionally, for non-left-censored methods, a much greater increase of NRMSE was observed when the MNAR rate increased from 50 to 80% than that from 20 to 50%, which was consistent with observations from the benchmark dataset (Figs. 2B, 5B).

**Figure 6:**
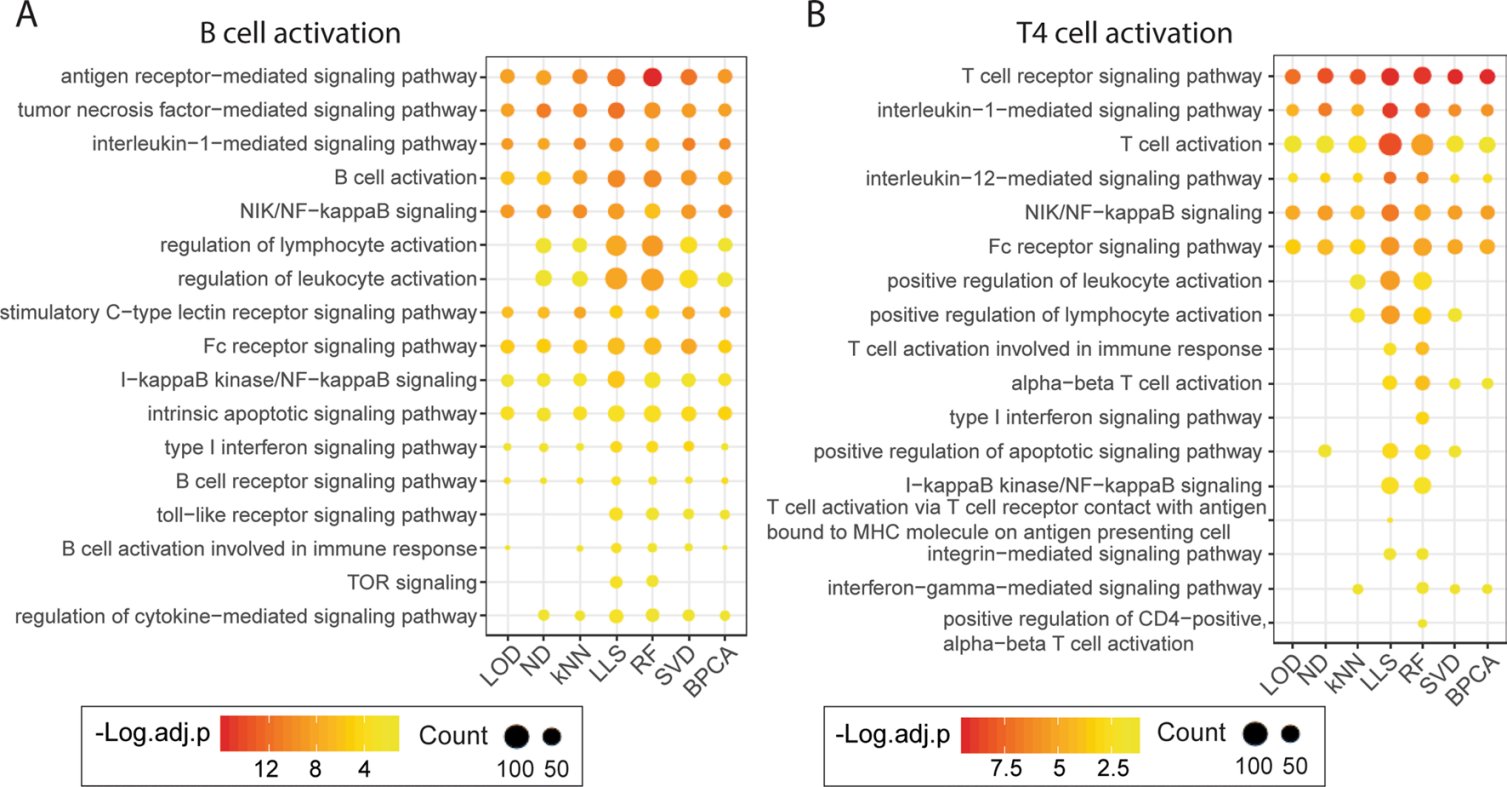
Enriched pathways of B cell and T4 cell activation with the immune cell dataset. Selected activation processes and signaling pathways that are significantly enriched (adjusted p-value < 0.05) in DE proteins of (**A**) B cell activation and (**B**) T4 cell activation. This figure was generated with R 3.6.1 (https://www.r-project.org) package ggplot2 v 3.2.1 (https://ggplot2.tidyverse.org).

The whole immune cell dataset containing 10,081 proteins and 27% missing values was imputed using different methods to test their performance on real missing values. For each immune cell type, DE proteins between activated and steady-state were determined by BH-adjusted p-value < 0.05. The over-represented biological processes from Gene Ontology (GO) annotation within the DE proteins were then determined by enrichment analysis and BH-adjusted p-value < 0.05 was considered as statistically significant. The enriched activation processes and signaling pathways used for comparison were selected based on the biological relevance to the specific stimulation of each cell type. Most B cell activation-related and monocyte activation-related pathways showed enrichment by all imputation methods but with different levels of significance (Figs. 6A, S3A). For example, when B cells were activated, imputation using LLS and RF resulted in more significant enrichment of regulation of lymphocyte activation, and more proteins related to this regulation process were characterized as DE proteins as well (Fig. 6A). Moreover, the TOR signaling pathway was essential in regulating B cell activation^19^, but significant enrichment of TOR signaling was only detected by RF and LLS imputation. The outperformance of RF and LLS was more apparent when comparing enriched pathways of activated T4 cells. T cell activation process was more significantly enriched by LLS and RF imputation, and more T cell activation DE proteins were detected as well (Fig. 6B). In addition, enrichment of T cell activation involved in immune response, I-kappaB kinase/NF-kappaB signaling and integrin-mediated signaling pathway were uniquely detected by RF and LLS imputation (Fig. 6B). For T8 cell activation, LLS, RF, and BPCA imputation could detect most of the relevant pathways. Notably, only the T cell activation process was enriched after LOD and ND imputation, and no pathway was significantly enriched with kNN imputation (Fig. S3B). Together, RF and LLS imputation consistently demonstrate the best performance in detecting enriched biological pathways in terms of the relevance to specific immune cell activations.

We further investigated whether specific gene signatures of T4 cell activation can be detected by different imputation methods. From a signature gene list of T4 cell activated by anti-CD3 and anti-CD28^20^, 21 genes were identified in the immune cell proteomic dataset with at least one missing value across all samples and detected as DE by at least one imputation method. Without imputation, eight proteins had sufficient values for statistical analysis and only one protein were determined DE protein in activated T4 cells. 14 and 15 DE proteins were detected by RF and LLS imputation, respectively, which markedly improved the detection of the signatures of T4 activation. Meanwhile, no obvious improvement was observed when imputation was performed using other methods (Fig. S5). These results suggested that RF and LLS outperformed other imputation methods at the individual protein level in terms of accurate detection of known gene signatures.

## Discussion

Missing value imputation is a key preprocessing step in large-scale label-free proteomic studies to improve proteome coverage and statistical power. A comprehensive evaluation of imputation methods is critical for selecting the most suitable one by understanding the advantages and disadvantages of each method. Our results revealed that the RF and LLS imputation methods consistently performed better than other methods, and RF slightly outperformed LLS in terms of protein ratio estimation and DE protein detection. Besides the consistently robust performance across all MV-MNAR situations with both datasets, RF imputation does not require parameter adjustment, together suggest that RF is the most suitable method for label-free proteomic studies without a comprehensive understanding of the mechanism of the missingness. Though RF imputation takes relatively longer time to process (Table S1), probably due to the intensive iterations of the algorithm, it’s considerably shorter than time-consuming steps in proteomics, such as data acquisition and database search, and could be shortened by high performance computers.

Performance of LLS imputation was highly comparable to RF-based on our results, however, when the MV rate and MNAR rate were both high, the intergroup protein ratios could be unreliable (Fig. 2D). In fact, the exceptionally high NRMSE of protein ratios was only observed in one dataset repeat in 30%MV-80%MNAR condition while NRMSE of protein abundances appeared normal. Though the reason for this phenomenon is not clear yet, it raises concern that protein ratios obtained after LLS imputation may be misleading.

Recent evaluation studies with metabolomic data showed contradictory results for SVD imputation^9,10^. Between the two global-structure imputation methods, our results have clearly shown that BPCA outperformed SVD, and the accuracy of SVD was greatly influenced by the high rate of MNAR. In addition, global structure methods are less preferable because of the high false discovery rate of DE protein detection, although BPCA achieved similar accuracy to RF in terms of NRMSE of protein intensities and protein ratios.

The proportion of MNAR missing values appears to be the primary determinant of imputation accuracy. Left-censored methods were devoted to handling MNAR as they replaced missing values with relatively small values. As expected, only left-censored methods showed improvement while the MNAR rate increased. On the other hand, it was surprising that the NRMSE of LLS, RF, SVD, and BPCA decreased slightly when the MV rate increased. This phenomenon was described for BPCA imputation with metabolomic data as well^10^. Contradictorily, Lazar et al. employed RMSE-observations standard deviation ratio (RSR), which was a normalized version of RMSE as well, for evaluation and demonstrated that performance of SVD imputation decreased with increasing MV rate in proteomic data^3^. Although how the MV rate affects imputation accuracy still needs further investigation, our results suggest that better imputation performance can be achieved by minimizing the MNAR rate.

If missing values in real datasets can be categorized into MAR and MNAR, it would be beneficial to apply imputation methods based on the mechanism of missingness. Left-censored methods, such as LOD or ND, can be used to impute MNAR missing values, and RF or LLS can be used to handle MAR missing values. This hybrid method handles every missing value individually based on its mechanism, which in theory provides the best estimation of missing values. However, missing values in real-world proteomic datasets are mixed MAR and MNAR that can hardly be characterized individually. For proteomic studies with multiple sample groups, particularly with different cell types, stimulations or drug treatments, it is common that certain proteins are only detectable in specific groups. For instance, the T-cell surface glycoprotein CD4 was completely missing in T8 cells, and the T-cell surface glycoprotein CD8B was completely missing in B cells and monocytes^14^. Therefore, if a protein is completely missing in one sample group, these missing values could be more likely MNAR. Previous studies have applied a similar method that one MV was considered MAR only if it appeared once in a sample group. Proteins with MAR missing values were filtered out from statistical analysis and MNAR missing values were replaced by zeros^21,22^. Nonetheless, whether the hybrid method is more accurate needs more investigation.

Our results of the enriched pathways of immune cell activation clearly showed that downstream analysis could be greatly impacted by imputation. For example, no pathway was significantly enriched for T8 cell activation if kNN imputation was applied (Fig. S3B). Interestingly, SVD and BPCA detected the largest amount of DE proteins in the benchmark dataset, but RF and LLS detected much more DE proteins than SVD and BPCA in the immune cell dataset (Fig. S4). This could be due to the SVD and BPCA algorithms are more robust with structured missing values, suggesting that RF and LLS perform better in handling real missing values. Analysis at the single protein level using signature genes of T4 cell activation further confirmed that RF and LLS detected DE proteins more accurately (Fig. S5), which could lead to better biological data interpretation.

There are some limitations in this study that need to be taken into consideration in future works. First, other preprocessing steps, such as log transformation, data filtering and normalization, were not considered in this study but would contribute to the overall accuracy of the data analysis framework. Thus, a more systematic comparison that combines all these steps will be highly desired. Second, we only imputed protein-level quantitative data for comparison. A previous study has indicated that imputation protein-level and peptide-level may result in different conclusions^3^, therefore, peptide-level data, as well as different approaches of aggregating peptide-level data to protein-level data, need to be assessed together with imputation methods. Third, we intentionally controlled the rates of MV and MNAR to simulate different mechanisms of missingness, however, the approach we applied tended to underestimate the MNAR rate. As MAR missing values were randomly selected after incorporating MNAR missing values, MNAR patterns could still be created by random chance, especially when the MV rate was high. Fourth, normalization is preferably performed before imputation^2^, thus an optimal normalization approach could improve the performance of RF imputation but needs further investigation.

The performance of imputation is greatly impacted by the rate and type of missing values in label-free proteomics. Proper selection of imputation methods markedly reduces error rate and improves downstream analysis. Our comparative study demonstrates that an RF-based imputation method has consistently the most robust performance and does not require a clear understanding of the mechanism of missingness, which is the most suitable for proteomic data. Our strategy to evaluate the detection of DE proteins and performance with real missing values can also be implemented in future studies.

## Methods

### Datasets

Two label-free proteomic datasets were used in this study. The first is a spike-in benchmark dataset where *E. coli* and yeast protein digest was mixed with human protein digest in different ratios. The second dataset is protein expression data of steady-state and activated immune cells.

The benchmark dataset contains seven groups with differential percentages of *E. coli* and yeast protein digest spiked in human protein digest. *Homo sapiens* colon cancer cell line SW620, *Saccharomyces cerevisiae* strain BY4741, and *E.coli* strain ATCC 25,922 were used to generate this dataset. We selected group A to D for this study. Each group has eight replicates in this dataset and the composition of *E.coli* and yeast proteins within a group are varied to mimic biological replicates (Table S2). Samples were analyzed by LC–MS and IonStar workflow as described previously^11^. Raw files and quantitative results are available on ProteomeXchange Consortium via PRIDE repository (https://www.ebi.ac.uk/pride/) with identifier PXD017915.

The immune cell dataset consists of protein intensities of seven cell lineages isolated from human blood^14^. Protein abundances were measured for both steady-state and activated states by the specific stimulus for each immune cell type. We selected the steady-state and activated data of B cells, T4 cells, T8 cells and monocytes in this study, which had four biological replicates (different blood donors) in each group, except that activated monocytes had three biological replicates. LFQ protein intensities were used as these values were more accurate^23^.

Intergroup protein ratios were calculated using Group A as the control for the benchmark dataset and the corresponding steady-state group as the control for each immune cell type. Adjusted p-values were calculated by two-sample equal variance Student’s t-test with Benjamini–Hochberg adjustment. An adjusted p-value lower than 0.05 was considered statistically significant.

### Missing value simulation

To incorporate missing values into the complete datasets with desired rates of MV and MNAR, we modified and implemented an approach described previously^3^. In brief, with α represents the rate of total missing values in the dataset D and β represents the ratio of MNAR missing values, if the dataset D contains N total values, N × α × β values will be replaced by missing values to simulate MNAR as follows: (1). A threshold matrix T with the same dimensions as D is generated from a normal distribution with µ = αth quantile of all values in D and σ = 0.3. (2). A probability matrix P with the same dimensions as D is generated from a Bernoulli distribution using β as the probability of success. (3). For each value D_i,j_ (i and j represents the ith row and jth column) in the complete dataset, if D_i,j_ < T_i,j_ and P_i,j_ = 1, D_i,j_ is selected as MNAR and replaced by a missing value. After MNAR incorporation, N × α × (1 − β) values are randomly chosen from the remaining values of the dataset and replaced by missing values to simulate MAR. Rows with only missing values were removed from the dataset.

### Missing value imputation

Instead of an exhaustive comparison, we picked two commonly used left-censored approaches: lowest of detection (LOD) and random drawing from a left-censored normal distribution (ND); three local similarity methods: k-nearest neighbors (kNN), local least squares (LLS), and random forest (RF); and two global-structure methods: Singular value decomposition (SVD) and Bayesian principal component analysis (BPCA). LOD imputation was performed by simply replacing missing values with the lowest value in the dataset. For ND imputation, missing values were replaced by random drew values from a normal distribution on the left tail of the abundance distribution of the complete dataset. The mean and standard deviation of this left-censored normal distribution was determined as: µ_i_ = µ_m_ − 2.2σ_m_ and σ_i_ = 0.3σ_m_ (µ = mean, σ = standard deviation, i = values for imputation, m = measured values). The parameters were identical to previously mentioned^14^. The kNN imputation was implemented by using the kNN function of the “VIM” R package^24^. LLS imputation was implemented by using llsImpute function of the “pcaMethods” package^25^. RF imputation was performed with missForest function of the “missForest” R package^16^. SVD and BPCA imputation were performed using pca function from the “pcaMethods” R package as well.

### Performance evaluation

Lower NRMSE indicates higher imputation accuracy. The nrmse function from “missForest” R package^16^was applied to calculate NRMSE of protein intensities and relative protein ratios between imputed and real datasets. In the benchmark dataset, yeast and *E. coli* proteins with adjusted p-values less than 0.05 were considered true positives (TP) and human proteins with adjusted p-values less than 0.05 were considered false positives (FP). Therefore, the false altered-protein discovery rate (FADR) was calculated as FADR = FP/(TP + FP). In the immune cell dataset, differentially expressed proteins (adjust p-value < 0.05) in the activated cells were used for the over-representation test of Gene Ontology (GO) biological processes. The analysis was conducted using “clusterProfiler” R package^26^.

## Supplementary Information

Supplementary Information.

## Data Availability

The benchmark proteomic dataset is available on ProteomeXchange Consortium via PRIDE repository (https://www.ebi.ac.uk/pride/) with identifier PXD017915. The immune cell dataset is available from Supplementary data of Rieckmann et al.^14^. All codes were written with R (Version 3.6.1), and R session information is available in Additional File 1. R codes are available at GitHub (https://github.com/liangjin0912/proteomics_imputation), and all packages used in this study are available at CRAN (https://cran.r-project.org/) or Bioconductor (https://www.bioconductor.org/).

## References

[1] Zhu W, Smith JW, Huang CM (2010). Mass spectrometry-based label-free quantitative proteomics. J. Biomed. Biotechnol..

[2] Karpievitch YV, Dabney AR, Smith RD (2012). Normalization and missing value imputation for label-free LC-MS analysis. BMC Bioinform..

[3] Lazar C, Gatto L, Ferro M, Bruley C, Burger T (2016). Accounting for the multiple natures of missing values in label-free quantitative proteomics data sets to compare imputation strategies. J. Proteome Res..

[4] Karpievitch Y (2009). A statistical framework for protein quantitation in bottom-up MS-based proteomics. Bioinformatics.

[5] Valikangas T, Suomi T, Elo LL (2018). A comprehensive evaluation of popular proteomics software workflows for label-free proteome quantification and imputation. Brief Bioinform..

[6] Webb-Robertson BJ (2015). Review, evaluation, and discussion of the challenges of missing value imputation for mass spectrometry-based label-free global proteomics. J. Proteome Res..

[7] Tuikkala J, Elo LL, Nevalainen OS, Aittokallio T (2008). Missing value imputation improves clustering and interpretation of gene expression microarray data. BMC Bioinform..

[8] Chiu CC, Chan SY, Wang CC, Wu WS (2013). Missing value imputation for microarray data: a comprehensive comparison study and a web tool. BMC Syst. Biol..

[9] Wei R (2018). Missing value imputation approach for mass spectrometry-based metabolomics data. Sci. Rep..

[10] Kokla M, Virtanen J, Kolehmainen M, Paananen J, Hanhineva K (2019). Random forest-based imputation outperforms other methods for imputing LC-MS metabolomics data: a comparative study. BMC Bioinform..

[11] Shen X (2018). IonStar enables high-precision, low-missing-data proteomics quantification in large biological cohorts. Proc. Natl. Acad. Sci. USA.

[12] Berg P, McConnell EW, Hicks LM, Popescu SC, Popescu GV (2019). Evaluation of linear models and missing value imputation for the analysis of peptide-centric proteomics. BMC Bioinform..

[13] Wang S (2020). NAguideR: performing and prioritizing missing value imputations for consistent bottom-up proteomic analyses. Nucleic Acids Res..

[14] Rieckmann JC (2017). Social network architecture of human immune cells unveiled by quantitative proteomics. Nat. Immunol..

[15] Kim H, Golub GH, Park H (2005). Missing value estimation for DNA microarray gene expression data: local least squares imputation. Bioinformatics.

[16] Stekhoven DJ, Buhlmann P (2012). MissForest-non-parametric missing value imputation for mixed-type data. Bioinformatics.

[17] Troyanskaya O (2001). Missing value estimation methods for DNA microarrays. Bioinformatics.

[18] Oba S (2003). A Bayesian missing value estimation method for gene expression profile data. Bioinformatics.

[19] Limon JJ, Fruman DA (2012). Akt and mTOR in B Cell activation and differentiation. Front. Immunol..

[20] Cao Y (2015). Functional inflammatory profiles distinguish myelin-reactive T cells from patients with multiple sclerosis. Sci. Transl. Med..

[21] Elo LL (2014). Statistical detection of quantitative protein biomarkers provides insights into signaling networks deregulated in acute myeloid leukemia. Proteomics.

[22] Foss EJ (2012). Proteomic classification of acute leukemias by alignment-based quantitation of LC-MS/MS data sets. J. Proteome Res..

[23] Cox J (2014). Accurate proteome-wide label-free quantification by delayed normalization and maximal peptide ratio extraction, termed MaxLFQ. Mol. Cell Proteom..

[24] Kowarik A, Templ M (2016). Imputation with the R Package VIM. J. Stat. Softw..

[25] Stacklies W, Redestig H, Scholz M, Walther D, Selbig J (2007). pcaMethods: A bioconductor package providing PCA methods for incomplete data. Bioinformatics.

[26] Yu G, Wang LG, Han Y, He QY (2012). clusterProfiler: an R package for comparing biological themes among gene clusters. OMICS.

