# Supplementary material for: A comparative study of evaluating missing value imputation methods in label-free proteomics
Source: Sci Rep. 2021 Jan 19;11:1760. doi: 10.1038/s41598-021-81279-4 (PMC7815892; doi:10.1038/s41598-021-81279-4)
Supplement: Supplementary file 1 — Supplementary Information. [file 41598_2021_81279_MOESM1_ESM.pdf]

A comparative study of evaluating missing value imputation methods in label-free proteomics

Liang Jin[1], Yingtao Bi[2], Chenqi Hu[1], Jun Qu[3,4], Shichen Shen[3,4], Xue Wang[1], Yu Tian[1,*]

Affiliations:

[1] Drug Metabolism and Pharmacokinetics, AbbVie Bioresearch Center, Worcester, Massachusetts 01605, United States

[2] Discovery and Exploratory Statistics, AbbVie Bioresearch Center, Worcester, Massachusetts 01605, United States

[3] Department of Pharmaceutical Science, SUNY at Buffalo, Buffalo, New York 14228, United States

[4] Center of Excellence in Bioinformatics& Life Science, Buffalo, NY 14203, United States

* Correspondence: yu.tian@abbvie.com

Supplementary information

Figure S1. Parameter optimization of imputation methods.

Figure S2. ROC curves of Group C-D/A with the benchmark dataset

Figure S3. Enriched pathways of monocyte and T8 cell activation with the immune cell dataset

Figure S4. Detection of DE proteins with the immune cell dataset

Figure S5. Differential expression of signature genes of T4 cell activation

Table S1. The average process time of imputation methods

Table S2. Detailed study design of the benchmark dataset

R session info

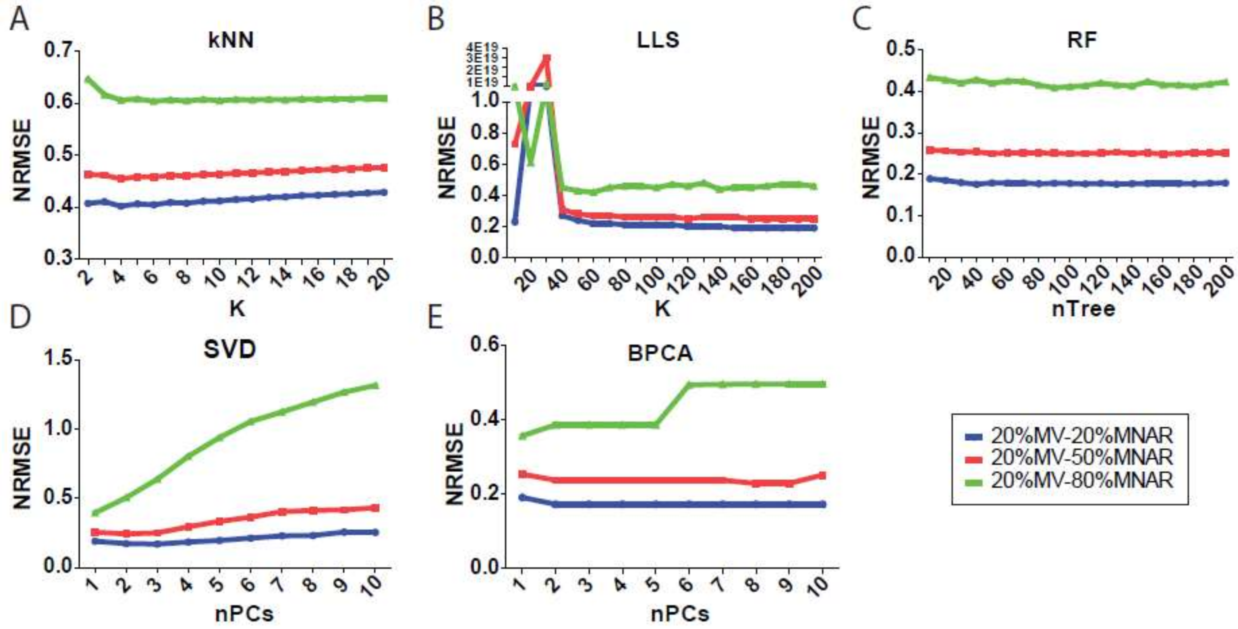

Figure S1. Parameter optimization of imputation methods. Selected parameters were tested for each imputation method using benchmark dataset containing 20% total missing values with 20-80% missing not at random (MNAR) rates. Parameters with the overall lowest normalized root mean square error (NRMSE) were considered optimal.

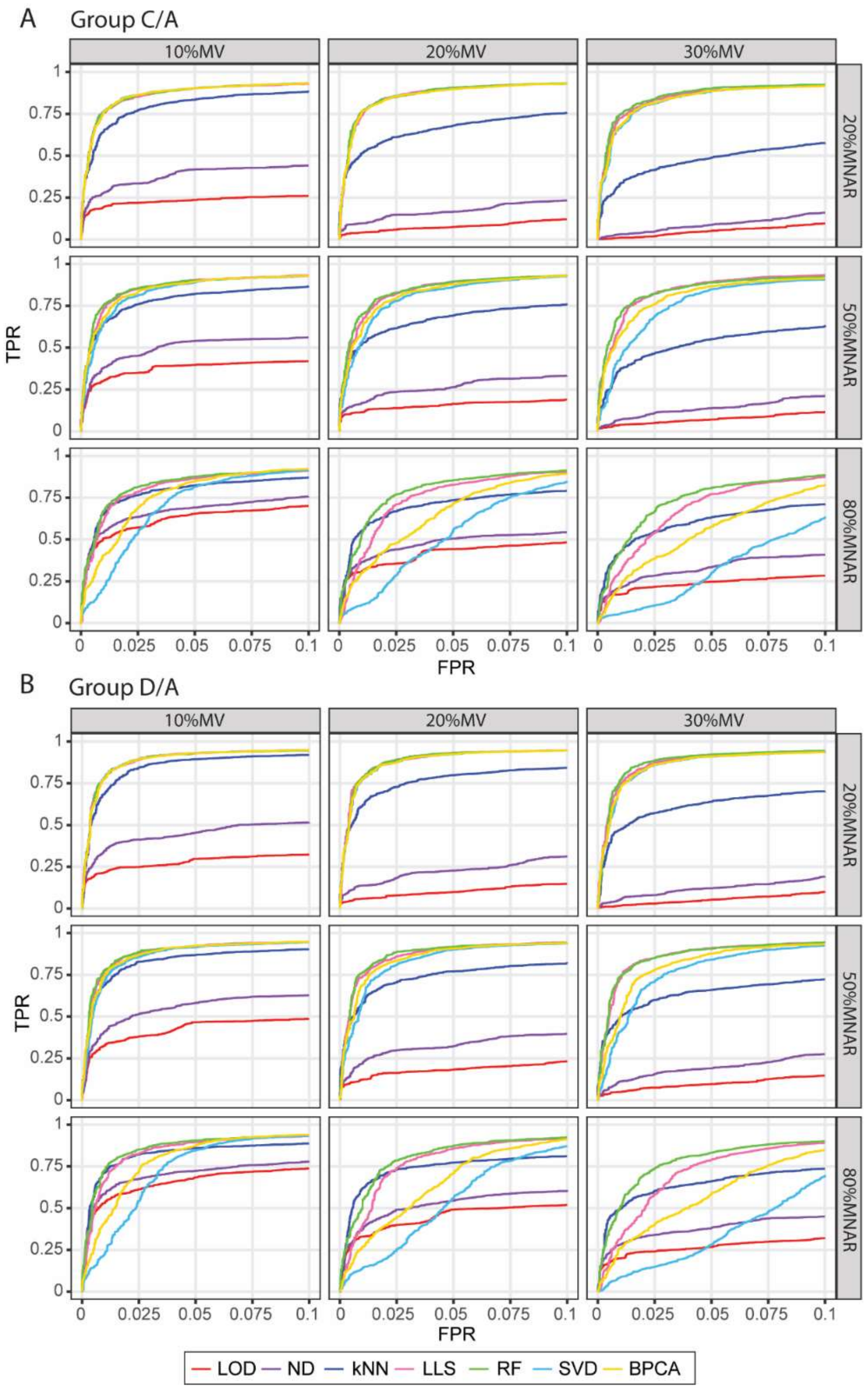

Figure S2. ROC curves of Group C-D/A with the benchmark datasets. ROC curves were plotted based on the average true positive rate (TPR) and false positive rate (FPR) of ten repeats in each MV-MNAR condition. *E.coli* and yeast proteins were considered true positives and human proteins are

considered false positives. This figure was generated with R 3.6.1 (https://www.r-project.org) package ggplot2 v 3.2.1 (https://ggplot2.tidyverse.org).

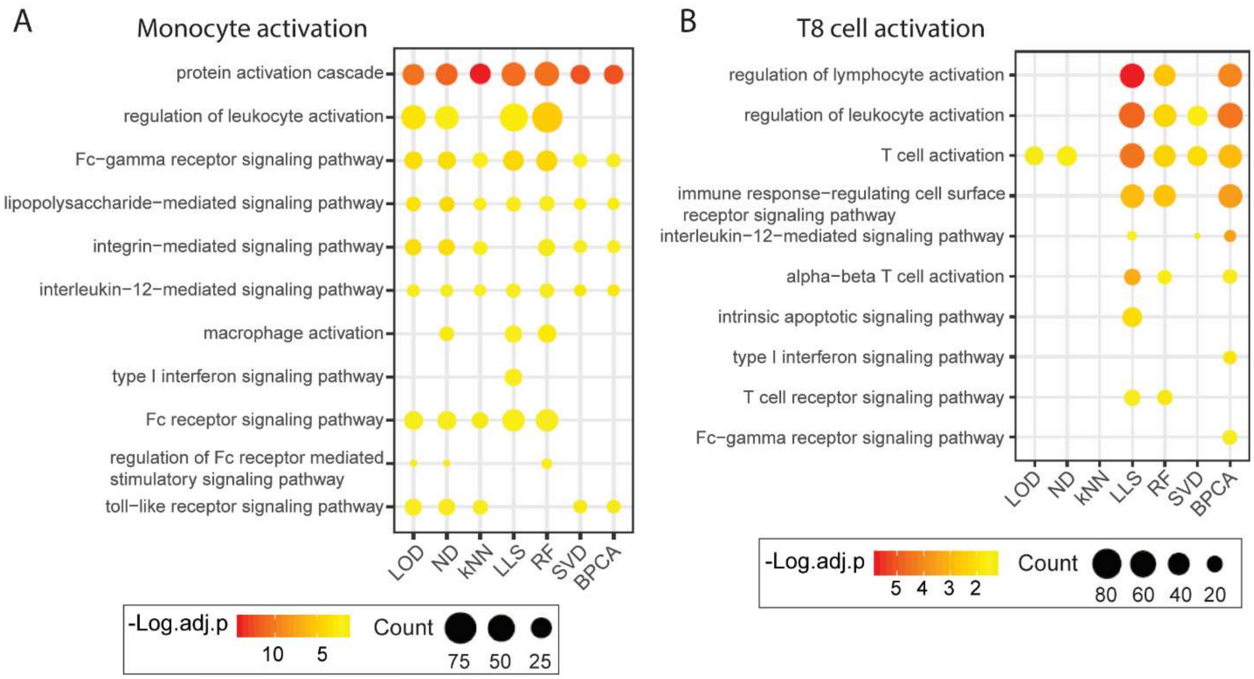

Figure S3. Enriched pathways of monocyte and T8 cell activation with the immune cell dataset. Selected activation processes and signaling pathways that are significantly enriched (adjusted p-value < 0.05) in DE proteins of (A) monocyte activation and (B) T8 cell activation. This figure was generated with R 3.6.1 (https://www.r-project.org) package ggplot2 v 3.2.1 (https://ggplot2.tidyverse.org/)

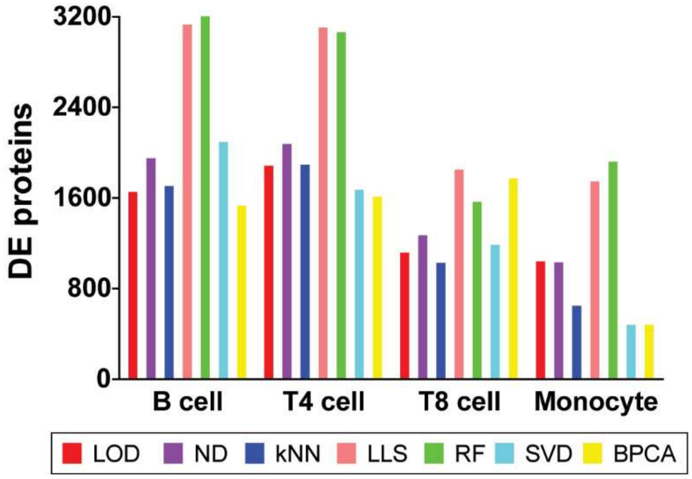

Figure S4. Detection of DE proteins with the immune cell dataset. Proteins with adjusted p-value < 0.05 between steady-state and activated cells were considered differentially expressed.

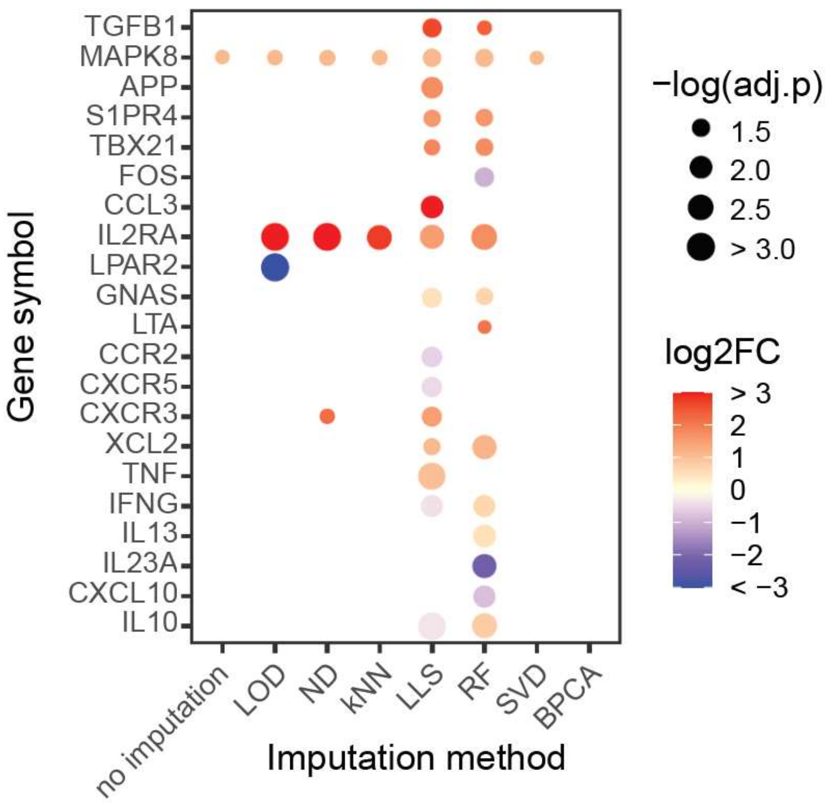

Figure S5. Differential expression of signature genes of T4 cell activation. 21 signature genes of T4 cell activated by anti-CD3 and anti-CD28 are differentially expressed with at least one imputation method. Dot size represents significance of differential expression (-log10 adjusted p-value), and color scale represents protein-level change of activated T4 cells vs. steady state T4 cells. This figure was generated with R 3.6.1 (https://www.r-project.org) package ggplot2 v 3.2.1 (https://ggplot2.tidyverse.org).

Table S1. The average process time of imputation methods.

Imputation method	Average process time of ninety benchmark datasets with incorporated missing values (second)	Average process time of ninety immune cell datasets with incorporated missing values (second)
LOD	~0	~0
ND	~0	~0
kNN	290.4	98.9
LLS	42.2	23.8
RF	2078.2	825.3
SVD	0.4	0.2
BPCA	45.6	20.1

Table S2. Detailed study design of the benchmark dataset.

Designated ratios (/Group A) of individual samples with intragroup variations								
Sample	A1	A2	A3	A4	A5	A6	A7	A8
E. coli	0.73	0.96	0.83	0.97	1.27	1.23	1.02	**1.00**
Yeast	1.05	1.01	1.03	1.01	0.95	0.95	1.00	**1.00**
Human	1.00	1.00	1.00	1.00	1.00	1.00	1.00	**1.00**
Sample	B1	B2	B3	B4	B5	B6	B7	B8
E. coli	1.55	1.88	1.56	1.17	1.23	1.31	1.80	**1.50**
Yeast	0.89	0.82	0.89	0.97	0.95	0.94	0.84	**0.90**
Human	1.00	1.00	1.00	1.00	1.00	1.00	1.00	**1.00**
Sample	C1	C1	C3	C4	C5	C6	C7	C8
E. coli	1.88	1.69	2.03	1.99	1.84	2.74	1.83	**2.00**
Yeast	0.82	0.86	0.79	0.80	0.83	0.65	0.83	**0.80**
Human	1.00	1.00	1.00	1.00	1.00	1.00	1.00	**1.00**
Sample	D1	D2	D3	D4	D5	D6	D7	D8
E. coli	2.77	2.78	1.71	2.24	2.67	2.83	2.50	**2.50**
Yeast	0.65	0.64	0.86	0.75	0.67	0.63	0.70	**0.70**
Human	1.00	1.00	1.00	1.00	1.00	1.00	1.00	**1.00**

Note: the bold number are theoretical intergroup protein ratios of each group/Group A.

R session information:

R version 3.6.1 (2019-07-05)

Platform: x86_64-w64-mingw32/x64 (64-bit)

Running under: Windows >= 8 x64 (build 9200)

Matrix products: default

locale:

[1] LC_COLLATE=English_United States.1252

[2] LC_CTYPE=English_United States.1252

[3] LC_MONETARY=English_United States.1252

[4] LC_NUMERIC=C

[5] LC_TIME=English_United States.1252

attached base packages:

```
 [1] grid      stats4    parallel  stats     graphics  grDevices
 [7] utils     datasets  methods   base
```

other attached packages:

```
 [1] openxlsx_4.1.0.1    tictoc_1.0          VIM_4.8.0
 [4] data.table_1.12.2   colorspace_1.4-1    pcaMethods_1.76.0
 [7] MSnbase_2.10.1      ProtGenerics_1.16.0 S4Vectors_0.22.1
[10] mzR_2.18.1          Rcpp_1.0.2          Biobase_2.44.0
[13] BiocGenerics_0.30.0 missForest_1.4      itertools_0.1-3
[16] iterators_1.0.12    foreach_1.4.7       randomForest_4.6-14
[19] ggplot2_3.2.1       dplyr_0.8.3
```

loaded via a namespace (and not attached):

```
 [1] vsn_3.52.0           carData_3.0-2
 [3] assertthat_0.2.1     BiocManager_1.30.4
 [5] sp_1.3-1             affy_1.62.0
```

```
 [7] cellranger_1.1.0      yaml_2.2.0
 [9] robustbase_0.93-5     impute_1.58.0
[11] pillar_1.4.2          backports_1.1.4
[13] lattice_0.20-38       glue_1.3.1
[15] limma_3.40.6          digest_0.6.21
[17] Matrix_1.2-17         preprocessCore_1.46.0
[19] plyr_1.8.4            MALDIquant_1.19.3
[21] XML_3.98-1.20         pkgconfig_2.0.3
[23] haven_2.1.1           zlibbioc_1.30.0
[25] purrr_0.3.2           scales_1.0.0
[27] ranger_0.11.2         affyio_1.54.0
[29] rio_0.5.16            BiocParallel_1.18.1
[31] tibble_2.1.3          IRanges_2.18.3
[33] car_3.0-4             withr_2.1.2
[35] nnet_7.3-12           lazyeval_0.2.2
[37] magrittr_1.5          crayon_1.3.4
[39] readxl_1.3.1          laeken_0.5.0
[41] ncdf4_1.16.1          doParallel_1.0.15
[43] MASS_7.3-51.4         forcats_0.4.0
[45] foreign_0.8-71        class_7.3-15
[47] tools_3.6.1           hms_0.5.1
[49] stringr_1.4.0         munsell_0.5.0
[51] zip_2.0.4             vcd_1.4-4
[53] compiler_3.6.1        e1071_1.7-2
[55] mzID_1.22.0           rlang_0.4.0
[57] rstudioapi_0.10       labeling_0.3
```

```
[59] boot_1.3-22         gtable_0.3.0
[61] codetools_0.2-16    abind_1.4-5
[63] curl_4.1            reshape2_1.4.3
[65] R6_2.4.0            zoo_1.8-6
[67] zeallot_0.1.0       stringi_1.4.3
[69] vctrs_0.2.0         lmtest_0.9-37
[71] DEoptimR_1.0-8      tidyselect_0.2.5
```
